# Yeast α-Glucosidase Inhibitory Phenolic Compounds Isolated from *Gynura medica* Leaf

**DOI:** 10.3390/ijms14022551

**Published:** 2013-01-28

**Authors:** Chao Tan, Qunxing Wang, Chunhua Luo, Sai Chen, Qianyuan Li, Peng Li

**Affiliations:** The First College of Clinical Medical Science, China Three Gorges University, Yichang 443003, Hubei, China; E-Mails: tanchao0125@sina.cn (C.T.); nchuayc@sina.cn (C.L.); csaiyc@sina.cn (S.C.); lqyuanyc@sohu.com (Q.L.); lpengyc@sohu.com (P.L.)

**Keywords:** *Gynura medica*, yeast α-glucosidase, flavonols, phenolic acids

## Abstract

*Gynura medica* leaf extract contains significant amounts of flavonols and phenolic acids and exhibits powerful hypoglycemic activity against diabetic rats *in vivo*. However, the hypoglycemic active constituents that exist in the plant have not been fully elaborated. The purpose of this study is to isolate and elaborate the hypoglycemic activity compounds against inhibition the yeast α-glucosidase *in vitro*. Seven phenolic compounds including five flavonols and two phenolic acids were isolated from the leaf of *G. medica*. Their structures were identified by the extensive NMR and mass spectral analyses as: kaempferol (**1**), quercetin (**2**), kaempferol-3-*O*-β-D-glucopyranoside (**3**), kaempferol-3-*O*-rutinoside (**4**), rutin (**5**), chlorogenic acid (**6**) and 3,5-dicaffeoylquinic acid methyl ester (**7**). All of the compounds except **1** and **3** were isolated for the first time from *G. medica*. Compounds **1**–**7** were also assayed for their hypoglycemic activity against yeast α-glucosidase *in vitro*. All of the compounds except **1** and **6** showed good yeast α-glucosidase inhibitory activity with the IC_50_ values of 1.67 mg/mL, 1.46 mg/mL, 0.38 mg/mL, 0.10 mg/mL and 0.53 mg/mL, respectively.

## 1. Introduction

*Gynura medica* is a recently newly found *Gynura* genus species belonging to the family of Compositae [1]. Previously, studies have demonstrated that the ethanol extract of *G. medica* showed good hypoglycemic activity in diabetic animal models [2,3]. However, the active constituents were not elucidated. *G. medica* extract also showed good antioxidant activity and some flavonols and phenolic acids were isolated or identified by HPLC-MS [4,5]. Many other plants of the genus of *Gynura* were found to inhibit the key enzymes relevant to type 2 diabetes (including α-glucosidase and α-amylase) and hypertension and show anti-diabetic and hypoglycemic activities [6–14]. The chemical constituents of the genus of *Gynura* included flavonoid, phenolic acid, cerebrosides, polysaccharide, alkaloids, terpenoids and sterols [15–19]. Phenolic acid, flavonoid and polysaccharide were the major hypoglycemic active components of *Gynura* genus.

Type 2 diabetes mellitus has become one of the world’s leading chronic diseases. Postprandial hyperglycemia was recognized as the characteristic for the type 2 diabetes. Medicinal plants were used for screening the anti-diabetic agents through varieties models *in vitro*, including inhibition of α-glucosidase and α-amylase [20–22], DPP-IV (dipeptidyl peptidase IV) [23], PTP-1B (Protein Tyrosine Phosphatases 1B) [24] and activation of PPAR-γ (peroxisome proliferator-activated receptor γ) [25]. Moreover, α-glucosidase was frequently used to screening the therapeutic agents for the control of postprandial hyperglycemia from the natural medicinal plants and isolated compounds.

Although previous studies have already demonstrated the *G. medica* extract showed good hypoglycemic activity *in vivo*, little information is available concerning the chemical constituents relevant to the hypoglycemic activity of the plant. The purpose of this study is to isolate and elaborate the hypoglycemic activity compounds against inhibition the yeast α-glucosidase *in vitro*.

## 2. Results and Discussion

**Figure 1:**
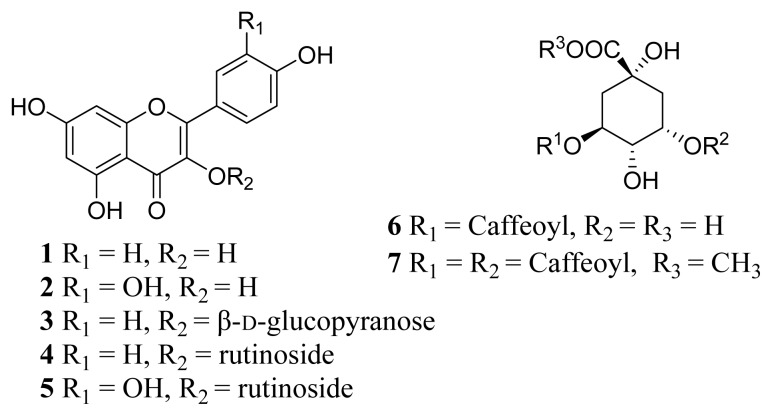
The chemical structures of the compounds **1**–**7** isolated from *G. medica* leaf.

Ethanol extract of *G. medica* leaf was successively fractionated with chloroform, ethyl acetate (EA) and *n*-butanol. All of the three organic extracts were evaluated their activity of inhibition the yeast α-glucosidase *in vitro*. Ethyl acetate (EA) extract showed the best activity against yeast α-glucosidase (data not shown) and was further isolated to get the active constituents. Seven phenolic compounds (Figure 1) including five flavonols and two phenolic acids were isolated and identified from the EA extract. Their structures were identified by the extensive NMR and mass spectral analyses.

Compound **1** was obtained as a yellow powder, the ESI-MS yielded a quasi-molecular ion peak [M–H] ^−^ at *m/z* 285.1. The UV spectrum showed λ_max_ at 265 nm and 367 nm. The ^1^H-NMR spectrum showed two peaks at δ 6.18 (1H, d, *J* = 1.8 Hz) and 6.42 ppm (1H, d, *J* = 1.8 Hz) consistent with the meta protons of flavonoid H-6 and H-8 on A-ring and an AA′BB′ system at δ 8.05 (2H, d, *J* = 8.9 Hz, H-2′, 6′) and 6.93 (2H, d, *J* = 8.9 Hz, H-3′, 5′) corresponding to the protons on B-ring. The MS and ^1^H-NMR data were compatible with those literatures of kaempferol [19]. Compound **2** was obtained as a yellow powder, the ESI-MS yielded a quasi-molecular ion peak [M–H]^−^ at *m/z* 301.0. The UV spectrum showed λ_max_ at 257 nm and 370 nm. The ^1^H-NMR spectrum showed two peaks at δ 6.18 (1H, d, *J* = 2.0 Hz) and 6.40 ppm (1H, d, *J* = 2.0 Hz) consistent with the meta protons of flavonoid H-6 and H-8 on A-ring and an ABX system at δ 7.67 (1H, d, *J* = 2.2 Hz, H-2′), 7.53 (1H, dd, *J* = 2.0 Hz, 8.4 Hz, H-6′) and 6.87 (1H, d, *J* = 8.4 Hz, H-5′). The MS and ^1^H-NMR data were compatible with those literatures of quercetin [19,26]. Compound **3** was obtained as a faint yellow powder, the ESI-MS yielded a quasi-molecular ion peak [M–H]^−^ at *m/z* 447.1. The UV spectrum showed λ_max_ at 265 nm and 346 nm. The ^1^H-NMR spectrum showed similar signal patterns to compound **1**, but the signal at δ 5.47 (1H, d, *J* = 7.2 Hz) followed by other characteristic additional signals indicate the presence of a sugar moiety in compound **3**. We carefully examined the ^13^C-NMR shift values of the sugar part in view of the reported literatures. It was suggested that, in order for it to be a glucopyranosyl unit, compound **3** was identified as kaempferol-3-*O*-β-d-glucopyranoside [27]. Compound **4** was obtained as a faint yellow powder, the ESI-MS yielded a quasi-molecular ion peak [M–H]^−^ at *m/z* 593.0. The UV spectrum showed λ_max_ at 265 nm and 345 nm. The ^1^H-NMR spectrum showed the similar signal patterns to compound **3**, a methyl signal 0.99 (3H, d, *J* = 6.2 Hz) in the high-field region was assigned to rhamnose. Compound **4** was suggested to be kaempferol-3-*O*-rutinoside [28]. Compound **5** was obtained as a faint yellow powder, the ESI-MS yielded a quasi-molecular ion peak [M–H]^−^ at *m/z* 609.0. The UV spectrum showed λ_max_ at 257 nm and 355 nm. The ^1^H-NMR spectrum showed two peaks at δ 6.20 (1H, d, *J* = 2.0 Hz) and 6.40 ppm (1H, d, *J* = 2.0 Hz) consistent with the meta protons H-6 and H-8 on A-ring and an ABX system at δ 7.54 (1H, d, *J* = 2.2 Hz, H-2′), 7.59 (1H, dd, *J* = 2.0 Hz, 9.0 Hz, H-6′) and 6.85 (1H, d, *J* = 9.0 Hz, H-5′). Compound **5** presented the same aglycone signal patterns of compound **2**, two anomeric proton signals at δ 5.32 (1H, d, *J* = 7.2 Hz) and 4.39 (1H, d, *J* = 1.6 Hz) were assignable to H-1 of a β-glucosyl proton and to the H-1 of a α-rhamnosyl proton, respectively. A methyl signal δ 0.99 (3H, d, *J* = 6.2 Hz) in the high-field region was assigned to rhamnose. Compound **5** presented the same glycoside signal patterns of compound **4**. Therefore, compound **5** was identified as rutin [28]. Compound **6** was obtained as a light yellow power. The ESI-MS yielded a quasi-molecular ion peak [M–H]^−^ at *m/z* 353.0. The UV spectrum showed λ_max_ at 327, 297 (shoulder) and 242 nm. The ^1^H-NMR spectrum showed a caffeoyl signals [δ 7.56 (1H, d, *J* = 15.9 Hz, H-7′), 7.05 (1H, brs, H-2′), 6.95 (1H, brd, *J* = 8.3 Hz, H-6′), 6.78 (1H, d, *J* = 8.3 Hz, H-5′), 6.27 (1H, d, *J* = 15.9, H-8′)] and a quinic acid signals [δ 5.34 (1H, m, H-5), 4.18 (1H, m, H-3), 3.75 (1H, dd, *J* = 8.1, 2.4 Hz, H-4), 2.20 (2H, m, H-2, H-6), 2.07 (2H, m, H-2, H-6)]. ^13^C-NMR spectrum showed 16 carbon signals as δ 74.7(C-1) 36.8(C-2) 69.9(C-3) 72.1(C-4) 70.5(C-5) 36.8(C-6) 175.6(C-7) 126.4(C-1′) 113.8(C-2′) 145.3(C-3′) 145.7(C-4′) 115.1(C-5′) 121.7(C-6′) 148.1(C-7′) 113.8(C-8′) 167.3(C-9′). Therefore, compound **6** was identified as chlorogenic acid [29]. Compound **7** was obtained as a yellowish amorphous powder, The ESI-MS yielded a quasi-molecular ion peak [M+Na]^+^ at *m/z* 487.1. The UV spectrum showed λ_max_ at 326, 298 (shoulder), 243 nm. The ^1^H-NMR spectrum showed similar signal patterns to compound **6**, but one more caffeoyl signal and a methoxyl were detected. The ^1^H-NMR spectrum showed two caffeoyl signals [δ 7.59, 7.62 (1H each, d, *J* = 15.9 Hz, H-7′, 7″), 7.07 (2H, brs, H-2′, 2″), 6.97 (2H, d, *J* = 8.1 Hz, H-6′, 6″), 6.79 (2H, d, *J* = 8.1 Hz, H-5′, 5″), 6.27, 6.35 (1H each, d, *J* = 15.9, H-8′, 8″)], a quinic acid signal [δ 5.43 (1H, m, H-5), 5.38 (1H, m, H-3), 3.99 (1H, m, H-4), 2.32–2.15 (4H, m, H-2, 6)] and a methoxyl signal [δ 3.98 (3H, s, OCH_3_)]. ^13^C-NMR signals as δ 73.1 (C-1), 34.4 (C-2), 71.0 (C-3), 70.5 (C-4), 71.1 (C-5), 34.5 (C-6), 126.4, 126.5 (C-1′, 1″), 113.7 (C-2′, 2″), 145.4, 145.5 (C-3′, 3″), 145.6, 145.9 (C-4′, 4″), 115.0 (C-5′, 5″), 121.5, 121.6 (C-6′, 6″), 148.1, 148.2 (C-7′, 7″), 113.6 (C-8′, 8″), 167.0, 167.5 (C-9′, 9″), 175.9 (COOCH_3_), 53.7 (OCH_3_). Therefore, compound **7** was identified as 3,5-dicaffeoylquinic acid methyl ester [29].

**Figure 2:**
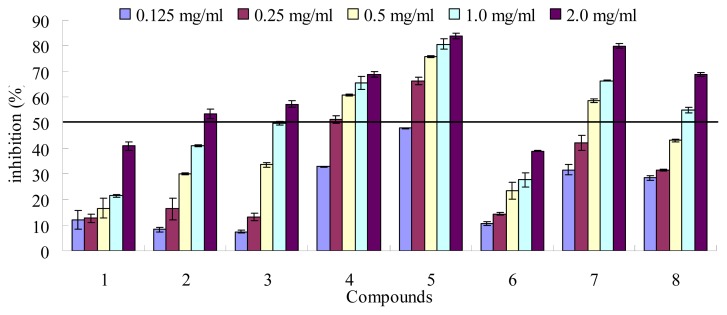
Yeast α-glucosidase inhibitory activity of the isolated compounds **1**–**7** and acarbose (**8**) as the control. Each value is mean ± standard derivation of three replicates.

Many Gynura species have recently been shown to exhibit hypoglycemic and carbohydrate enzyme inhibitory activities [11–14]. The inhibition of yeast α-glucosidase activity of the extracts and pure phenolic compounds **1**–**7** were determined. The clinical anti-diabetic drug acarbose (**8**) was used as a reference (Figure 2 and Table 1). All of the compounds showed yeast α-glucosidase inhibitory activity in a dose-dependent manner (Figure 2). Except compounds **1** and **6**, others showed more than 50% inhibitory activity at 2.0 mg/mL concentrations. The IC_50_ values were calculated (Table 1). Compounds **4**, **5** and **7** showed the best activity with the IC_50_ values of 0.38 mg/mL, 0.10 mg/mL and 0.53 mg/mL, respectively. While the clinical anti-diabetic drug acarbose (**8**) showed moderate activity *in vitro* with the IC_50_ values of 0.99 mg/mL, which was consistent with literature [29]. Compounds **2** and **3** also showed somewhat activity against yeast α-glucosidase.

*G. segetum* and *G. divaricata* has recently been shown to exhibit two key enzymes relevant to type 2 diabetes including α-glucosidase and α-amylase [12–14]. It was reported that the flavonoids compounds maybe responsible for the α-glucosidase inhibitory activity. However, the active constituents were unknown [13]. An activity-guided phytochemical isolation method was used to study the active compounds in *G. medica*. Both crude extracts of ethyl acetate and *n*-buthanol were all showed α-glucosidase inhibitory activity compared with the positive durg acarbose (data not shown). However, the ethyl acetate extract showed better activity than *n*-buthanol extract. Therefore, the further isolation was conducted on the ethyl acetate extract. Seven phenolic compounds including five flavonols and two phenolic acids were isolated. Flavonol and its glycosides (**2**–**5**) and the dicaffeoylquinic acid methyl showed good activity, which was in good agreement with previous reports that many flavonoids from plants have been reported as α-glucosidase inhibitors [29,30].

## 3. Experimental Section

### 3.1. Plant Material

*Gynura medica* was obtained in July of 2010 from Huoshan districts, Anhui province, China. A voucher specimen (2010R01) was deposited at the pharmacy of The First College of Clinical Medical Science, China Three Gorges University. The *G. medica* leaves were dried at room temperature for three weeks and finely powdered in a knife mill.

### 3.2. General Experimental Procedures

^1^H and ^13^C**-**NMR data were recorded on a Bruker Avance-600 FT NMR spectrometer with TMS as internal standard. Electrospray Ionization Mass Spectral (ESI-MS) data were obtained on a Q-Star Elite mass spectrometer equipped with a Turbo Ionspray source. Analytical and semi-preparative High performance liquid chromatography (HPLC) was performed on a Shimadzu LC-20 HPLC system. Column chromatography was carried with silica gel (200–300 mesh), RP-ODS and Sephadex LH-20 (18–110 μm) were obtained from Pharmacia Co. α-glucosidase (yeast, EC 3.2.1.20) powder, acarbose and 4-nitrophenyl-α-d-glucopyranoside (pNPG) were purchased from Sigma-Aldrich (St. Louis, MO, USA). All other solvents were analytical grade and were purchased from Sinopharm Chemical Reagent Co., Ltd (Shanghai, China).

### 3.3. Extraction of *G. medica* Leaf

The weighed portion of the crude drug 2 kg was extracted twice with 80% ethanol (*v*/*v*) under reflux at 80 °C. The extract was evaporated to dryness *in vacuo*. The dry ethanol extract was successively fractionated with chloroform, ethyl acetate (EA) and *n*-butanol, respectively. The yields of those three organic extracts were 31.2 g, 56.5 g and 89.5 g, respectively. All of the extracts were evaluated according to their activity of inhibition the yeast α-glucosidase *in vitro*. The EA fraction was further chromatographed on a Silica gel column eluted with mixture of chloroform and methanol to afford six sub-fractions (Fr.A_1_–A_6_). Fr.A_2_ was further chromatographed on a Sephadex LH-20 column eluted with mixture of chloroform-methanol (1:1) to yield compound **1** (15 mg) and **2** (5.8 mg). Fr.A_3_ and Fr.A_5_ was further chromatographed on a Sephadex LH-20 column eluted with methanol and next on a RP-ODS column or semi-preparative HPLC to give compound **3** (11 mg), **4** (7.9 mg), **5** (8.2 mg), **6** (5.5 mg) and **7** (4.5 mg).

### 3.4. Yeast α-Glucosidase Inhibitory Assay

Yeast α-glucosidase inhibitory activity was determined as described in the literature [29]. Briefly, a mixture of 50 μL of different concentrations of the samples and 100 μL of 0.1 M phosphate buffer (pH 6.9) containing yeast α-glucosidase solution (1.0 U/mL) were incubated in 96 well plates at 25 °C for 10 min. After pre-incubation, 50 μL of 5 mM pNPG solution in 0.1 M phosphate buffer (pH 6.9) was added to each well at timed intervals. The reaction mixturses were incubated at 25 °C for 5 min. Absorbance was recorded with a micro-plate reader (Multiskan MK3) at 405 nm before and after incubation with pNPG solution and compared to that of the control which had 50 μL buffer solutions instead of test samples. The yeast α-glucosidase inhibitory activity was expressed as inhibition % and was calculated as follows:

(1) $$\text{inhibition}\%=100\times [(C_{5}-C_{0})-(S_{5}-S_{0})]/(C_{5}-C_{0})$$

Where *C*_0_ and *C*_5_ were the OD values of control (buffer solutions instead of test samples) at 0 min and 5 min at 405 nm, respectively. *S*_0_ and *S*_5_ were the OD values of samples at 0 min and 5 min at 405 nm, respectively.

## 4. Conclusions

Seven phenolic compounds were isolated from the ethyl acetate extract of the leaf of *G. medica*. Their structures were identified as kaempferol (**1**), quercetin (**2**), kaempferol-3-*O*-β-d-glucopyranoside (**3**), kaempferol-3-*O*-rutinoside (**4**), rutin (**5**), chlorogenic acid (**6**) and 3,5-dicaffeoylquinic acid methyl ester (**7**). All of the compounds except **1** and **3** were isolated from *G. medica* for the first time. All of the compounds except **1** and **6** were showed good yeast α-glucosidase inhibitory activity. Compounds **4**, **5** and **7** showed promising activity with the IC_50_ values of 0.38 mg/mL, 0.10 mg/mL and 0.53 mg/mL, respectively.

## Figures and Tables

**Table 1:** The IC_50_ values of yeast α-glucosidase inhibitory activity of the isolated compounds **1**–**7** and acarbose (**8**) as the control. Each value is mean ± standard derivation of three replicates.

Compounds	IC_50_ (mg/mL)
1	>2.0
2	1.67 ± 0.05
3	1.46 ± 0.03
4	0.38 ± 0.03
5	0.10 ± 0.01
6	>2.0
7	0.53 ± 0.02
8	0.99 ± 0.02
